# Transcript variants of long-chain acyl-CoA synthase 1 have distinct roles in sheep lipid metabolism

**DOI:** 10.3389/fgene.2022.1021103

**Published:** 2022-11-22

**Authors:** Yang Cao, Yongsheng Yu, Lichun Zhang, Yu Liu, Kaizhi Zheng, Sutian Wang, Haiguo Jin, Lixiang Liu, Yang Cao

**Affiliations:** ^1^ Institute of Animal Biotechnology, Jilin Academy of Agricultural Science, Gongzhuling, China; ^2^ Institute of Animal Husbandry and Veterinary, Zhejiang Academy of Agricultural Sciences, Hangzhou, China; ^3^ Institute of Animal Science, Guangdong Academy of Agricultural Sciences, Guangzhou, China

**Keywords:** ACSL1, transcript variants, sheep, APA, lipid metabolism

## Abstract

Mutton has recently been identified to be a consumer favorite, and intermuscular fat is the key factor in determining meat tenderness. Long-chain acyl-CoA synthetase 1 (ACSL1) is a vital subtype of the ACSL family that is involved in the synthesis of lipids from acyl-CoA and the oxidation of fatty acids. The amplification of the ACSL1 gene using rapid amplification of cDNA ends revealed that the alternative polyadenylation (APA) results in two transcripts of the ACSL1 gene. Exon 18 had premature termination, resulting in a shorter CDS region. In this study, the existence of two transcripts of varying lengths translated normally and designated ACSL1-a and ACSL1-b was confirmed. Overexpression of ACSL1-a can promote the synthesis of an intracellular diglyceride, while ACSL1-b can promote triglyceride synthesis. The transfection of ACSL1 shRNA knocks down both the transcripts, the triglyceride content was significantly reduced after differentiation and induction; and lipidome sequencing results exhibited a significant decrease in 14–22 carbon triglyceride metabolites. The results of the present study indicated that the ACSL1 gene played a crucial role in the synthesis of triglycerides. Furthermore, the two transcripts involved in various interactions in the triglyceride synthesis process may be the topic of interest for future research and provide a more theoretical basis for sheep breeding.

## 1 Introduction

Sheep are widely reared as herbivorous livestock all over the world. Mutton is a high protein and low cholesterol meat, which contains more Ω-3 polyunsaturated fatty acids, monounsaturated fatty acids, single-chain fatty acids, and conjugated linoleic acid (Chikwanha et al., 2017). These fatty acids are associated with human health and can provide essential fatty acids in a healthy human diet, which has been welcomed by consumers in recent years. Intermuscular fat is the key factor in determining meat tenderness and is the primary component of marbling in meat products that consumers appreciate (Crouse et al., 1984). However, excess fat accumulation will result in feed waste and animal burden. Therefore, a better understanding of adipose tissue metabolic rules can improve production efficiency.

Long-chain acyl-CoA synthetases (ACSLs) are crucial for the synthesis of acyl-CoA while ACSLs can activate fatty acids guiding them into specific metabolic pathways (Ellis et al., 2010). The catalyzed acyl-CoA was involved in different pathways; however, there is no evidence to prove that acyl-CoA is linked to a specific pathway. ACSL1 and ACSL5 both are involved in the production of acyl-CoA, which is primarily used in triglyceride synthesis and mitochondrial oxidation. The ACSLs are saturated and unsaturated fatty acids with 8–22 carbon triglycerides, in addition; each subtype has a different substrate preference. The first one, ACSL1, prefers saturated and monounsaturated fatty acids with 16–18 carbon triglycerides (Suzuki et al., 1990). The subtypes of ACSLs exist with specific abundances in different tissues. ACSL1 is more abundant in the liver and adipose tissues (Paul et al., 2014), and the ester acyl-CoA is essential for triglyceride synthesis (Pan et al., 2010). ACSL1 overexpression was found to promote oleic acid infiltration into triglycerides in NIH-3T3 fibroblasts and PC12 neurons (Souza et al., 2002; Marszalek et al., 2004). Post-overexpression of ACSL1 in mice hearts resulted in a 12-fold increase in triglyceride content and a 1.5-fold increase in choline glycerin phospholipid content (Chiu et al., 2001). During 3T3-L1 adipocyte differentiation, a four-fold increase was observed in the ACSL1 expression, in addition to this, no change was found in the expression levels of other subtypes indicating ACSL1 as the major player in promoting 3T3-L1 adipocytes’ triglyceride synthesis (Yue, 2013). Previous studies mentioned that the increase in the activity of acyl-CoA can be achieved by overexpressing ACSL1 in primary hepatocytes of rats. However, ACSL1 increased oleic acid infiltration into phospholipids and diglycerides, but did not promote oleic acid infiltration into triglycerides (Gluchowski et al., 2017). ACSL1 contains multiple promoters, consequently resulting in multiple transcripts with distinct functions in different tissues (Suzuki et al., 1995). The different transcripts of the ACSL1 gene may be responsible for the various roles of the ACSL1 gene in triglyceride synthesis.

Alternative polyadenylation (APA) is important in regulating gene stability, translation, and transport (Sandberg et al., 2008; Mayr and Bartel, 2009). In addition to playing a regulatory role in the gene expression process, APA can result in transcripts with varying lengths of 3′UTR. APA is a ubiquitous phenomenon in transcription that has been discovered in more than 50% of human and 30% of mice genes (Tian et al., 2005). APA regulates gene expression, thereby regulating specific biological functions. APA is classified into two types among which the first one includes the normal termination of transcription. Polyadenylation occurs at the 3′end. Multiple polyA sites are located downstream of the stop codon, resulting in the formation of transcripts with different lengths encoding the same protein, this type of 3′UTR is considered a tandem 3′UTR. The second one occurs at different poly-A sites located in different exons or introns, resulting in coding regions of varying lengths that encode different proteins (Sun et al., 2012).

Previous experiments compared the production performance of Duhan hybrid sheep (Dorper sheep × Small-tailed Han sheep F1) and Small-tailed Han sheep and observed the significantly higher average daily gain of hybrid sheep than that of Small-tailed Han sheep, whereas the comparatively increased intramuscular fat and oleic acid were also reported for hybrid sheep. Using MeDIP-Seq (methylated DNA immunoprecipitation sequencing), two populations of Duhan sheep and Small-tailed Han sheep were sequenced for whole-genome methylation. The results demonstrated a significant difference between expression levels of ACSL1 when two different groups were analyzed (*p* < 0.05), and the ACSL1 gene is regulated by methylation, which affects lipid metabolism and meat quality (Cao et al., 2017). Although the sheep ACSL1 gene sequence is unclear, the existence of multiple transcript variants within this gene is an intriguing area to investigate further.

## 2 Materials and methods

### 2.1 Materials

The tissues used for the experiment were isolated from 40-day-old Small-tailed Han sheep raised by the Jilin Academy of Agricultural Sciences. Also, the cells were stored in the Jilin Academy of Agricultural Sciences.

### 2.2 Rapid amplification of cDNA ends

Primers were designed according to the principles of rapid amplification of cDNA ends (RACE) primer design using ACSL1 gene sequences from GenBank. Primer Premier 6.0 software was used to design 3′UTR primers and labeled as SP1, SP2, SP3, and SP4 (Table 1).

**TABLE 1:** Sequence of primers.

Primer name	Sequence/(5′-3′)	Product size/bp
SP1	CCT​TGG​CAG​CCA​GAT​AAT​TCA	450 bp
SP2	GGA​CAA​GCA​AAC​ACC​ACG​CTG​A	
SP3	CTG​GCA​CAA​GGG​GAG​TAC​ATA​GCT​CC	750 bp
SP4	TTC​GGA​ATT​ATT​TCA​GGT​CAC​AGA​TCG​ATG	1,570 bp
ACSL1-a-F	ATG​ATG​CAA​GCC​CAC​GAG​CTG​T	2,100 bp
ACSL1-a-R	TTA​GAC​TTT​GAT​GGT​GGA​GTA​A	
ACSL1-b-F	ATGATGCAAGCCCACGAG	1,836 bp
ACSL1-b-R	TCAGGCACCTAGTGTCT	
NB-F	CCC​TTG​TTT​AGG​CTC​TCG​G	471 bp
NB-R	TTG​GGC​TTC​TGT​CTG​TTG​G	
ACSL1-YF1	GCC​ATC​ACC​TAC​ATC​ATC​AAC​AA	151 bp
ACSL1-YR1	ACA​CTT​CTT​GCC​TCG​TTC​CA	
ACSL1-YF2	CAG​AAA​CAA​GGA​TGT​CAA​AAA​A	120 bp
ACSL1-YR2	GAG​TTC​AGG​GTG​GAG​ATA​GAT​G	
β-actin-F	GTCCACCTTCCAGCAGAT	96 bp
β-actin-R	GCT​AAC​AGT​CCG​CCT​AGA​A	
PPARγ-F	GAGCTGACCCGATGGTT	150 bp
PPARγ-R	TGAGGGAGTTGGAAGGC	
CEBPα-F	CGT​GGA​GAC​GCA​ACA​GAA​G	105 bp
CEBPα-R	AAGATGCCCCGCAGTGT	
LPL-F	GCC​AAA​AGA​AGC​AGC​AGC​AAG	178 bp
LPL-R	GCA​GGG​TAA​AAG​GGA​TGT​T	

Reverse transcription, 5′-end phosphorylation of primers, and ligation assays were performed according to modified protocols of the manufacturers of SMARTer RACE Kit (Clontech). Tissue RNA was extracted and reverse transcribed with specific primers followed by nested PCR with the primers listed in Table 1.

The target fragment was amplified according to the RACE system. PCR reaction cycles are as follows: 94°C for 30 s, 72°C for 3 min, five cycles; 94°C for 30 s, 70°C for 30 s, 72°C for 3 min, five cycles; 94°C for 30 s, 68°C for 30 s, 72°C for 3 min, 25 cycles. After ligating with pmd18-t vector, the fragment was sequenced by a biological company.

Based on the RACE result, primers F1, R1, F2, and R2 (Table 1) were used to clone the CDS regions of two different transcripts of the sheep ACSL1 gene. ACSL1-a was amplified with primers F1 and R1, while amplification of ACSL1-b was carried out with F2 and R2. A biological company sequenced the target fragment that had been ligated with the pmd18-t vector.

### 2.3 Northern blotting

Comparing the sequences of the two transcripts led to the selection of the common sequence as the target. Probe primers (NB-F and NB-R in Table 1) were designed using the software Primer Premier 6.0.

The probes were prepared using PCR with a DIG marker. Isolation of total RNA from the heart, liver muscle, and adipocytes was carried out *via* TRIzol. After 1% formaldehyde denaturing gel electrophoresis, the membrane was transferred using the upward capillary method for 20 h and then fixed at 80°C for 2 h. The denatured probe was used for overnight hybridization at 50°C after a 2 h prehybridization. The membrane was rinsed at room temperature after hybridization, and the machine was exposed for 3 h to detect the signal.

### 2.4 Western blotting

The cells were digested with trypsin and then collected after a short centrifugation. Lysis buffer (RIPA, Thermo Scientific) was added to the lysate cells and protein concentration was determined using an Enhanced BCA Protein Assay Kit (Beyotime). In a proportionally diluted sample, 5 × loading buffer was added. Denaturation was carried out at 98°C for 10 min using 10% SDS-PAGE gel electrophoresis, and then 80 V electrophoresis for 30 min was performed which was switched to 120 V electrophoresis for 2 h. In an ice box, the film was transferred for 90 min at 200 mA. The polyvinylidene difluoride (PVDF) membranes were washed thrice with TBST (Tris-buffered saline with 0.1% Tween 20 detergent) and then blocked with 5% skimmed milk at room temperature for 2 h. The membranes were then incubated with a primary antibody. The corresponding species were incubated with the secondary antibody solution at room temperature for 2 h. The film was then rinsed with TBST thrice and the strip was detected using an ECL-PLUS kit (Beyotime).

### 2.5 Real-time PCR

Primers were designed to detect transcript expression in two sheep tissue samples based on the CDS region derived from a cloned ACSL1 gene. The other primers were designed based on sequences on the NCBI (PPARγ ID: XM_012099243.2, C/EBPα ID:NM_001308574.1, LPL ID:NM_001009394.1).

Total RNA from the heart, liver, spleen, lung, kidney, intestine, adipose, and muscle was extracted using the TRIzol method and reverse transcribed into cDNA (Takara). Quantitative real-time PCR (qRT-PCR) amplification was performed with SYBR Premix Ex Taq using Roche 480 LightCycler. The target gene expression was analyzed using the 2^−ΔΔCt^ method and normalized to β-actin.

### 2.6 Immunofluorescence

Preadipocytes were sown in a 24-well plate (10^4^ cells per well) for cell culturing and counting. The next day, a 10-fold increase in mitochondrial dye was applied to preadipocytes. Fluorescence was detected using a fluorescence microscope after 16 h. After fixing the cells at room temperature for 20 min, the fixative solution was rinsed and sealed with the blocking solution for 15 min. The primary antibody (ACSL1, Bioss) was diluted in the proportion of 1:500 and further incubated at 4°C overnight. The cells were kept at room temperature for 1 h the next day before being rinsed three times with phosphate-buffered saline (PBS). The secondary antibody (anti-rabbit, CST) was diluted in 1:1,000 proportions and further incubated at room temperature for 2 h. The cells were then washed thrice with PBS. DAPI (4,6-diamidino-2-phenylindole) was diluted in proportion and stained at room temperature for 15 min. Superfluous dye was rinsed away before examining the fluorescence under the microscope.

### 2.7 Bioinformatics analysis

ORF finder (http://www.ncbi.nlm.nih.gov/gorf/orfig.cgi) was used to determine the coding region of the gene, UCSC (http://genome.ucsc.edu/) was utilized to analyze the exon, prediction of the subcellular location of the protein was conducted using PSORT II (https://www.genscript.com/tools/psort). Protean (DNASTAR) was used to predict the secondary structure of the ACSL1 protein, while the tertiary structure of the ACSL1 protein was predicted *via* SWISS-MODEL (http://swissmodel.expasy.org/).

### 2.8 Cell transfection and sample collection

The shACSL1-297 (GGG​CAT​ACA​GGT​GTC​CAA​TAA) was synthesized and ligated to the pGPU6 vector for sequencing. The recombinant shRNA vector (1 μg) of ACSL1 was transfected into 1 × 10^6^ preadipocytes using FuGENE (omega). On day 8, fully differentiated cells that had matured into adipocytes were collected. In the lipid metabolome analysis, at least 10^7^ cells were used. The trypsinized cells were then transferred into an EP tube. The supernatant was removed, and the cells were rinsed with pre-cooled PBS and stored at −80°C.

#### 2.8.1 Oil Red O staining

The mature adipocytes were washed three times with PBS and subjected to fixation in 4% paraformaldehyde followed by staining with 60% Oil Red O solution (Sigma).

### 2.9 Sequencing and data analysis

All data analysis processes were based on the self-built database MWDB (Metware Biotechnology Co., Ltd. Wuhan, China).

The intracellular lipids were extracted and analyzed by the liquid chromatography–mass spectrometry (LC–MS) system (Chen, 2009).

Consideration was given to metabolites with a variable important in projection (VIP) values of ≥1 and having fold changes ≥2 or fold changes ≤0.5. Using the R package MetaboAnalystR, VIP values were extracted from the OPLS-DA (orthogonal projections to latent structures discriminant analysis) data, which also included score plots and permutation plots.

Identified metabolites were annotated using the KEGG Compound database (http://www.kegg.jp/kegg/compound/), and annotated metabolites were then mapped to the KEGG Pathway database (http://www.kegg.jp/kegg/pathway.html). Pathways with significantly regulated metabolites were then fed into MSEA (Metabolite Set Enrichment Analysis), and their significance was determined by hypergeometric test’s *p*-values.

### 2.10 Statistical analyses

Three independent biological experiments were conducted and the corresponding mean values were calculated. All data are reported as the mean ± SEM, and all statistical analyses were carried out using the *t*-test. Differences with a value of *p* < 0.05 were considered statistically significant.

## 3 Results

### 3.1 Cloning and validation of the ACSL1 gene

**FIGURE 1:**
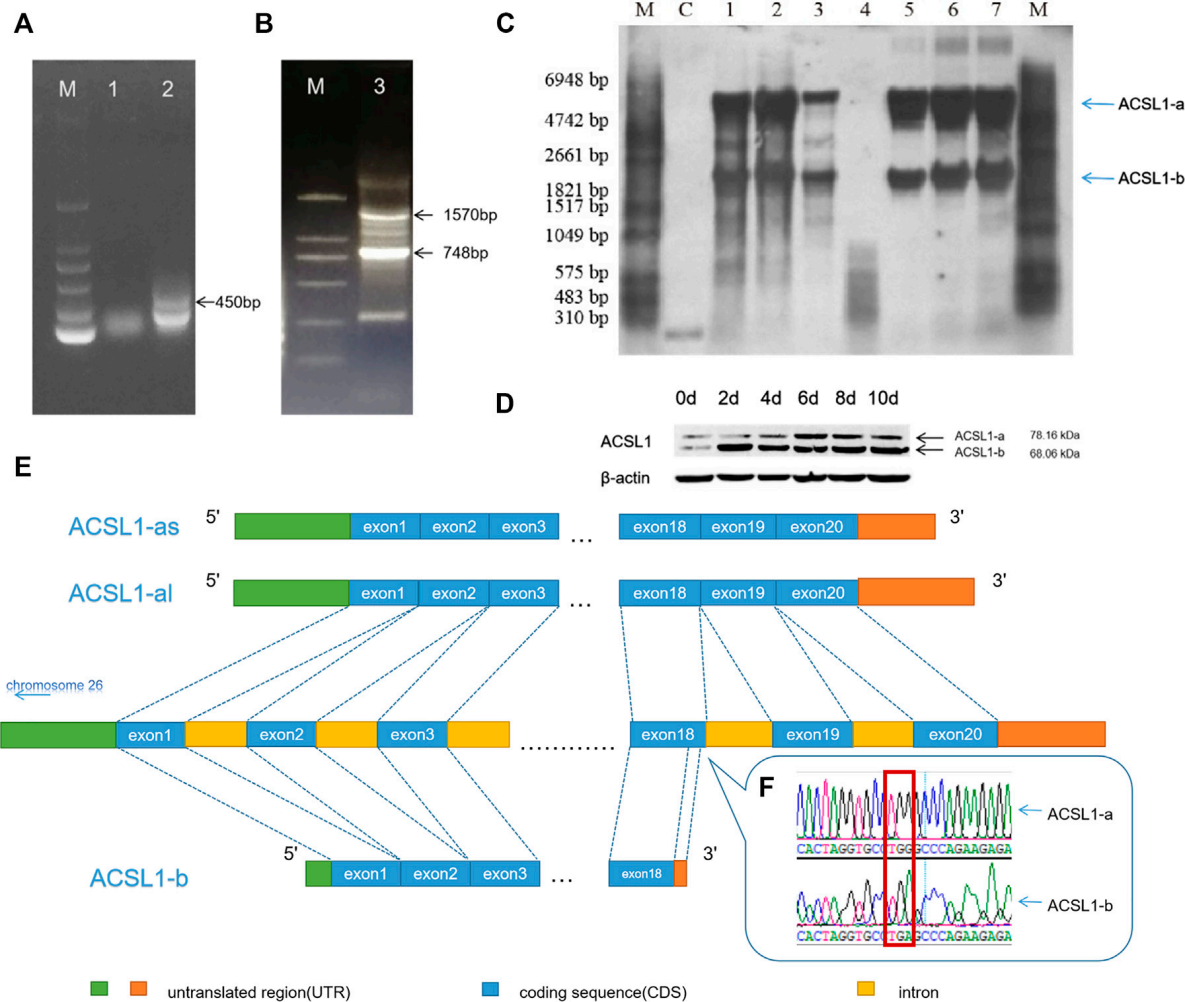
**(A)** 3′RACE PCR result of the sheep ACSL1 gene of SP1 and SP2. **(B)** 3′RACE PCR result of the sheep ACSL1 gene of SP3 and SP4 (lane M: DL2000 marker; lane 1: 3′RACE by SP1; lane 2: 3′RACE by SP2; lane 3: 3′RACE by SP3 and SP4). **(C)** Northern blotting results of sheep tissues and adipocytes of different differentiation stages (lane M: RNA molecular weight marker; lane C: positive control; lane 1: heart; lane 2: liver; lane 3: muscle; lane 5: sheep preadipocytes on the day 1 of differentiation; lane 6: sheep preadipocytes on the day 4 of differentiation; lane 7: the induced differentiation of sheep preadipocytes on day 8). **(D)** Western blotting results of sheep tissues and adipocytes of different differentiation stages (when cells were added to the induction medium, it was taken as day 0). **(E)** Schematic diagram of different transcripts of the ACSL1 gene. **(F)** RACE sequencing results of G–A mutation.

The primers were designed using the RACE primer design principle for 3′RACE PCR, and fragments with the 450 bp length were obtained using primers SP1 and SP2 (Figure 1A). The sequencing results revealed a G–A mutation site in the cloned sequence. The G–A mutation led to premature termination of the ACSL1 mRNA translation pathway. This would result in the formation of a short ACSL1 protein.

Amplification of the CDS region downstream with primers SP3 and SP4 produced distinct lengths of 3′UTR (748 bp and 1,579 bp) (Figure 1B), resulting in the CDS region having two different lengths (2,100 bp and 1,836 bp). Therefore, there are two different length CDS regions (2,100 bp and 1,836 bp) for ACSL1.

The results were further verified using Northern blotting which was used to detect the mRNA expression of ACSL1 gene transcripts in different tissues and stages of sheep preadipocyte differentiation (Figure 1C). The known sequence was used for the designing of primers. The 471 bp long probe was located at 320 bp in the CDS region. The results demonstrated that there are at least two different transcripts in the heart, liver, and muscle tissues. Preadipocyte differentiation in sheep involves two transcripts at different stages, and the expression of ACSL1 gene mRNA was found to be increasing during adipocyte differentiation.

The expression of two transcripts of the ACSL1 gene in sheep preadipocytes at different stages of differentiation stages was detected using Western blotting (Figure 1D). The proteins of ACSL1 have molecular weights of 78.16 and 68.06 kDa.

Northern and Western blotting results revealed the presence of at least two transcripts of the ACSL1 gene. Therefore, the transcript with a 2,100 bp long CDS region is defined as ACSL1-a, along with the other transcript ACSL1-b of 1,836 bp length. ACSL1-a has two distinct 3′UTRs. Genome sequence alignment revealed the presence of 21 exons in ACSL1-a, while ACSL1-b terminated early at exon 18 also these two transcripts share the same region before exon 18 (Figures 1E,F).

ACSL1-a protein levels gradually increased during preadipocyte differentiation, reaching their peak on the day 6. ACSL1-b protein also increased in the preadipocytes and reached the highest levels on the day 2.

### 3.2 Bioinformatics analysis and expression of the ACSL1 gene in sheep

**FIGURE 2:**
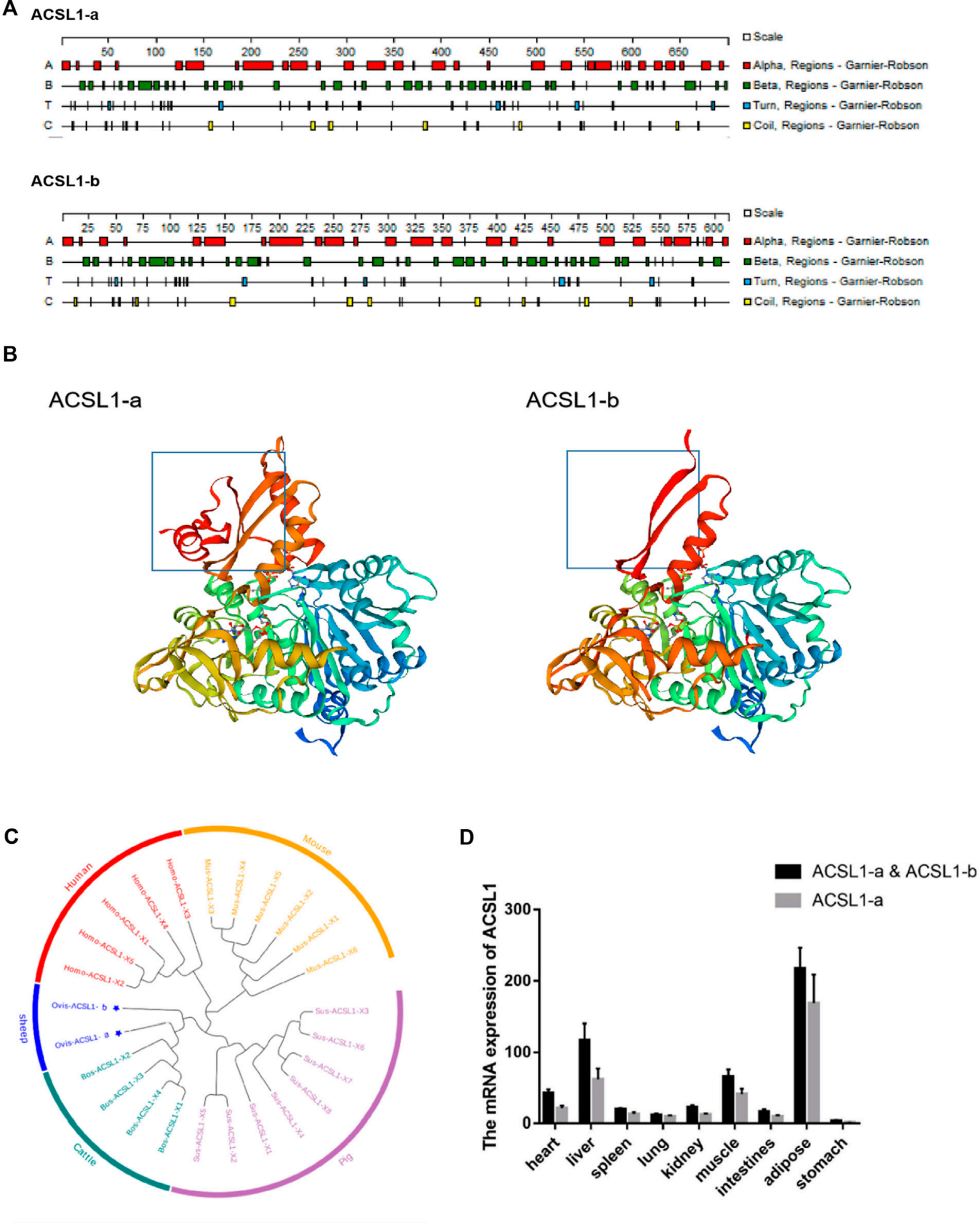
**(A)** Secondary structures of ACSL1-a and ACSL1-b proteins. **(B)** Tertiary structures of ACSL1-a and ACSL1-b proteins. **(C)** Phylogenetic tree depicting different ACSL1 transcripts. **(D)** Expression result of ACSL1 gene expression in various tissues.

Sheep ACSL1-a encodes 699 amino acids with a protein of molecular weight of 78.16 kDa; however, ACSL1-b encodes 611 amino acids with a protein having a molecular weight of 68.06 kDa. A comparison of the amino acid sequences of the transcripts revealed the absence of 88 amino acids in ACSL1-b with 86.98% similarity among amino acid sequences. ACSL1-a and ACSL1-b have protein instability coefficients of 34.25 and 34.17, respectively, and are both stable proteins. Protean was used for the prediction of secondary structures of two transcripts of the sheep ACSL1 gene. There are 32 *α*-helices, 47 *β-*corners, 35 t-corners, and 29 irregular curls in the ACSL1-b protein whereas ACSL1-b protein includes 27 *α*-helices, 41 *β*-corners, 32 t-corners, and 27 irregular curls (Figure 2A). The secondary structure folds into the tertiary structure, along with the prediction of ACSL1 protein by SWISS-MODEL is depicted in the figure (Figure 2B).

The sheep ACSL1 transcript was compared to human, mouse, pig, and cattle transcripts available on NCBI. The sheep ACSL1 gene transcript demonstrated higher homology with the cattle ACSL1 gene transcript which was consistent with the law of evolution (Figure 2C). The expression levels of different ACSL1 transcripts in 40 day-old sheep were analyzed and the results demonstrated a similarity between the expression of ACSL1-a and ACSL1-b in different tissues. The expression level of ACSL1 was the highest in adipose tissue, followed by the liver, muscle, heart, kidney, duodenum, spleen, lung, and the lowest expression level was found in the stomach (Figure 2D).

**FIGURE 3:**
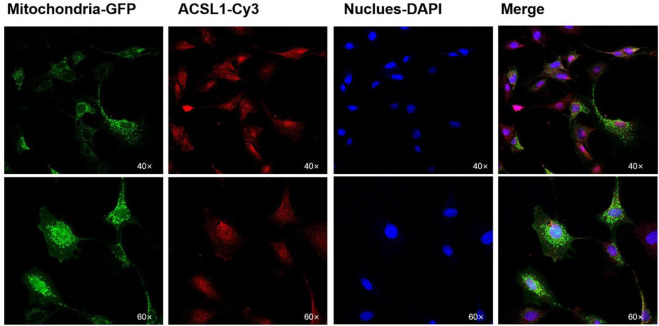
Subcellular localization of ACSL1 in sheep preadipocytes.

PSORT was used to predict the subcellular localization of the ACSL1 gene in cells, and ACSL1-a was found to be located in mitochondria, nucleus, and cytoplasm; however, ACSL1-b protein was identified in mitochondria and nucleus (Table 2). Laser confocal imaging revealed that the ACSL1 gene expression was observed in both mitochondria and nucleus, which was consistent with the online prediction (Figure 3).

**TABLE 2:** Prediction of sheep ACSL1 subcellular localization.

Location	ACSL1-a (%)	ACSL1-b
Mitochondrial	52.2	69.6%
Nuclear	43.5	30.4%
Cytoplasmic	4.3	—

### 3.3 Overexpression of ACSL1-b in sheep adipocytes increased the triglyceride content

Previously, researchers from our laboratories reported that there was no substantial increase in the total triglyceride content in the cell after ACSL1-a overexpression. Lipidome sequencing, verification, and overexpression of ACSL1-a can enhance the synthesis of diglycerides as well as certain triglyceride metabolites. These results suggested that the major function of ACSL1-a might be associated with the boosting of diglycerides synthesis (Cao et al., 2020).

**FIGURE 4:**
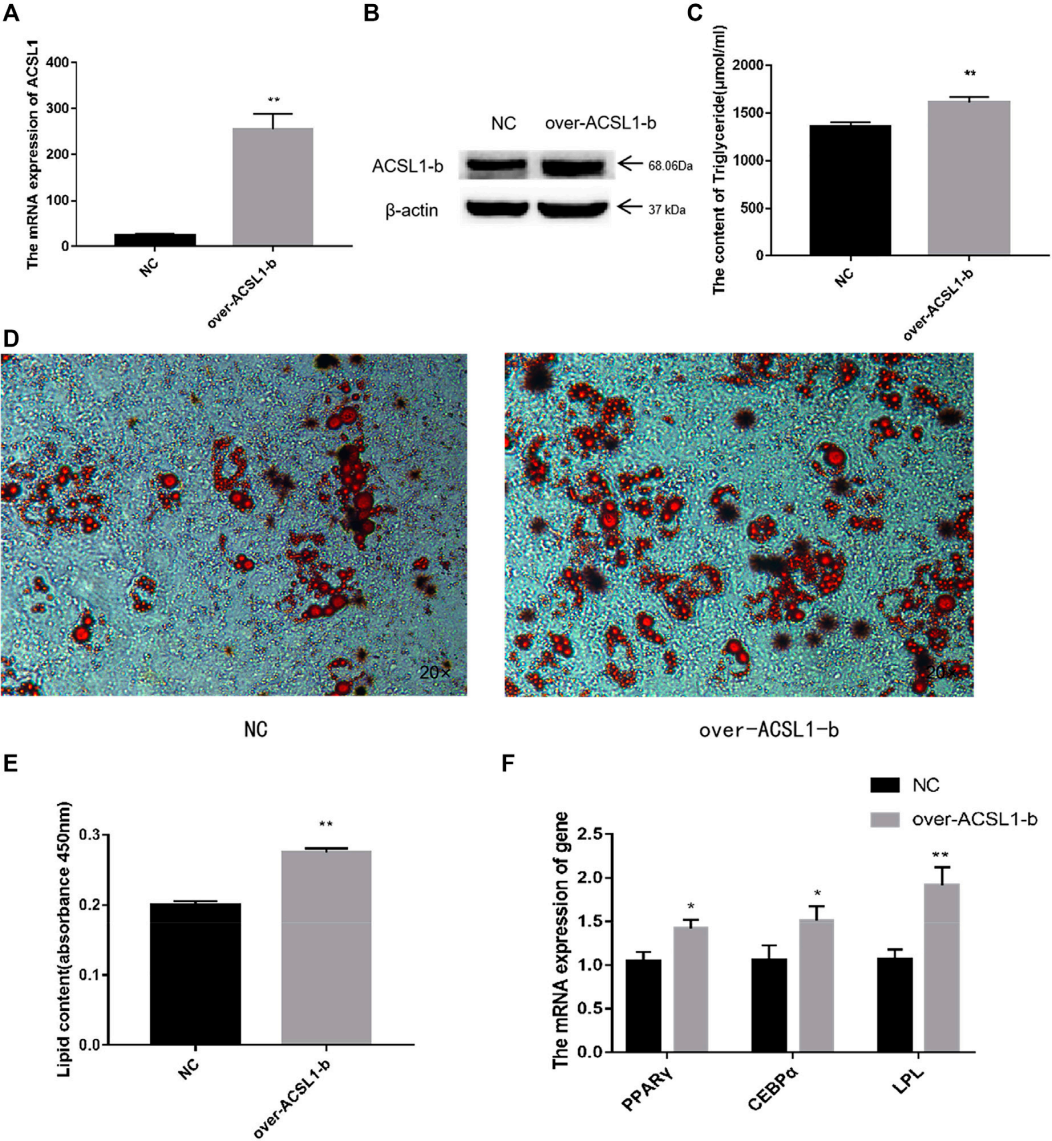
Overexpression of the ACSL1-b in sheep preadipocytes: **(A)** mRNA expression of the ACSL1-b on day 8 of differentiation (*p < 0.05, **p < 0.01). **(B)** Protein expression of the ACSL1-b on day 8 of differentiation. **(C)** Detection of intracellular triglyceride content. **(D)** Oil Red staining of adipocytes after induced differentiation on day 8 (left: control; right: overexpression ACSL1-b). **(E)** Absorbance value of Oil Red O after extraction. **(F)** mRNA expression of PPARγ, CEBPα, and LPL in sheep adipocytes. NC: adipocytes transfected with the pBI-CMV3 vector for control. Over-ACSL1: adipocytes transfected with the pBI-CMV3 vector with ACSL1-b CDS.

The CDS region of the ACSL1-b gene was cloned and ligated into the pBI-CMV3 vector, and the vectors were further transfected into sheep preadipocytes. ACSL1-b mRNA and protein expression in sheep adipocytes increased significantly on day 8 after of differentiation induction (Figures 4A,B). The transfected cells further developed into mature adipocytes.

The total TGA content analysis of adipocytes from the overexpression group was significantly higher than that of the control group (Figure 4C). Furthermore, the number of lipid droplets generated in the ACSL1-b overexpression group was significantly higher than that of the control group (Figure 4D). The Oil Red O in the cells was extracted for absorbance detection. The OD value of the ACSL1-b overexpression group was significantly higher than that of the control (Figure 4E). The mRNA expression of PPARγ, CEBPα, and LPL in sheep adipocytes also increased significantly (Figure 4F).

### 3.4 Lipidome analysis of metabolites post ACSL1 gene knockdown

**FIGURE 5:**
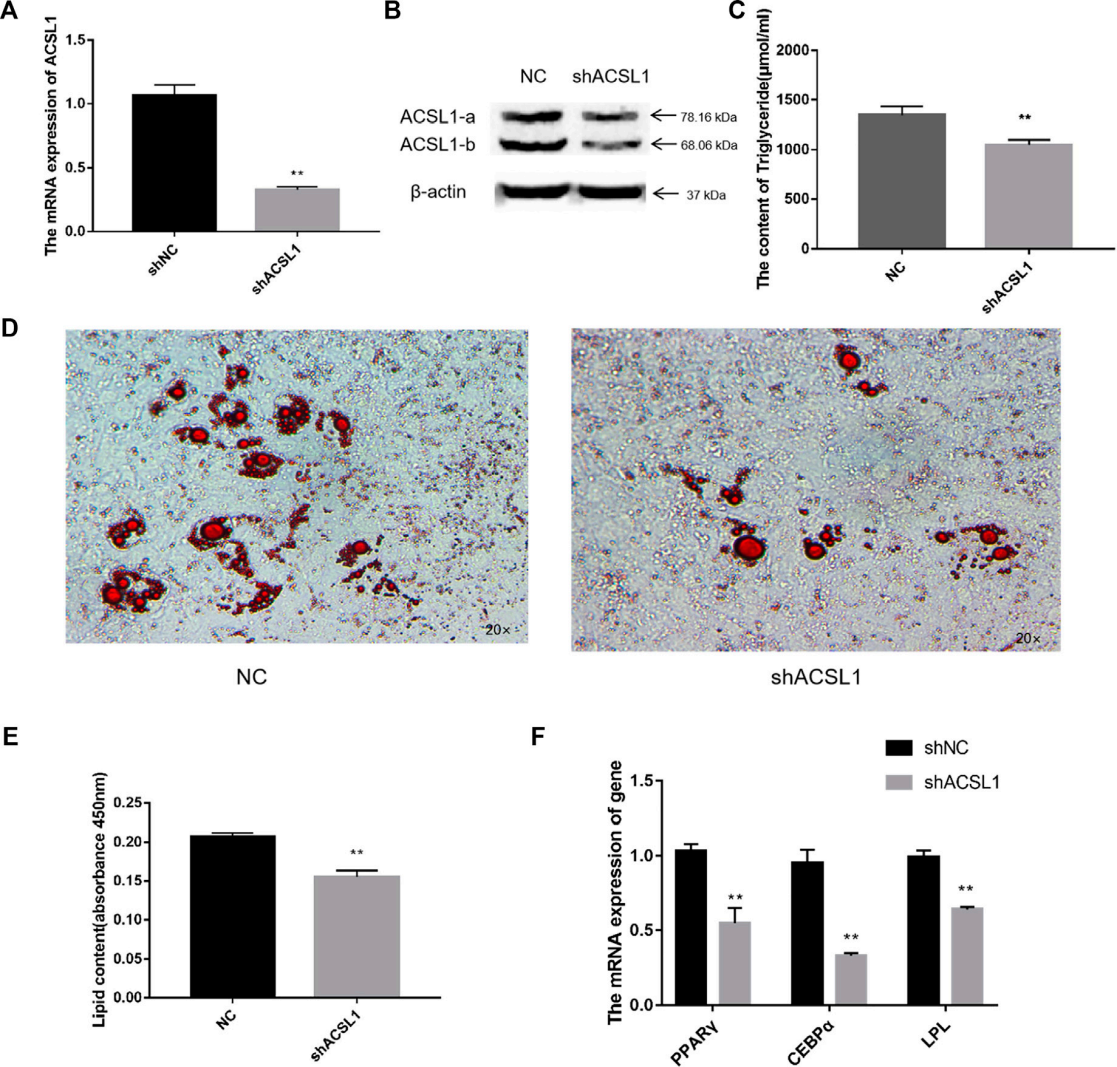
Knocked down of the ACSL1 gene in sheep preadipocytes: **(A)** mRNA expression of the ACSL1 gene on day 8 of differentiation (*p < 0.05, **p < 0.01). **(B)** Protein expression of the ACSL1 gene on day 8 of differentiation. **(C)** Detection of intracellular triglyceride content. **(D)** Oil Red staining of adipocytes after induced differentiation on day 8 (left: control; right: knocked down ACSL1). **(E)** Absorbance value of Oil Red O after extraction. **(F)** mRNA expression of PPARγ, CEBPα, and LPL in sheep adipocytes. NC: adipocytes transfected with shNC for control. ShACSL1: adipocytes transfected shRNA-297 of ACSL1 gene.

To interfere with the expression of ACSL1, shRNA targeting a common sequence using both the ACSL1 transcripts was designed. The mRNA and protein levels of both transcripts were dramatically reduced after the interference (Figures 5A,B), similarly, the triglycerides and lipid droplets in the cells also reported a significant reduction (Figures 5C–E). The mRNA expression of PPARγ, CEBPα, and LPL in sheep adipocytes reduced significantly (Figure 5F). Lipidome sequencing was used in this work to detect alterations in cells.

**FIGURE 6:**
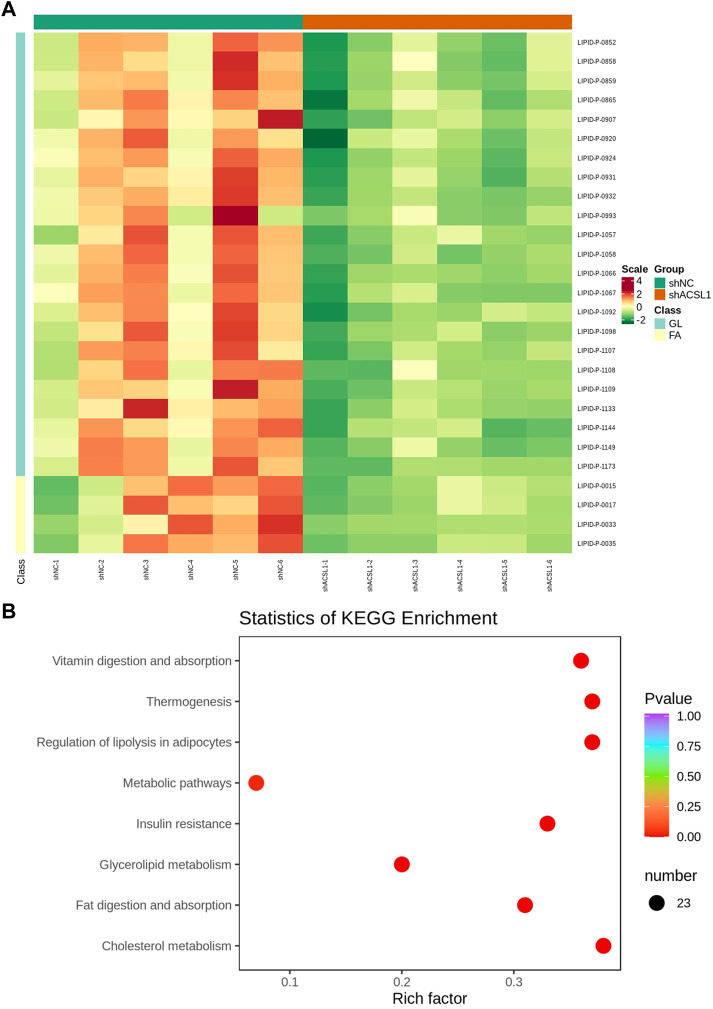
Lipid metabolome sequencing: **(A)** heat map; **(B)** KEGG analysis. shNC: adipocytes transfected with shNC as control. ShACSL1: adipocytes transfected shRNA-293 of the ACSL1 gene.

The metabolome contained 469 metabolites, 27 of which were differential metabolites, with four acylcarnitine metabolites and 23 triglycerides metabolites shown to be downregulated (Figure 6A; Table 3). The amount of 14–22 carbon triglyceride metabolites and acylcarnitine metabolites, such as lauroyl-carnitine, myristoyl-carnitine, tetradecenoyl-carnitine, and palmitoleoyl-carnitine decreased significantly.

**TABLE 3:** Differential metabolites in adipocytes knocking down the ACSL1 gene compared with the controls.

Compound	Class	Fold change	Type
Lauroyl-carnitine	CAR	0.381792978	Down
Myristoyl-carnitine	CAR	0.400777014	Down
Tetradecenoyl-carnitine	CAR	0.206371467	Down
Palmitoleoyl-carnitine	CAR	0.314336933	Down
TG (16:0/16:1/20:1)	TG	0.48022308	Down
TG (16:0/16:1/22:1)	TG	0.498540902	Down
TG (14:0/20:1/20:1)	TG	0.433355204	Down
TG (14:0/20:1/22:1)	TG	0.481539957	Down
TG (16:0/16:1/18:2)	TG	0.453217617	Down
TG (18:0/18:1/18:2)	TG	0.448890681	Down
TG (14:0/20:1/20:2)	TG	0.360978679	Down
TG (18:1/18:2/20:0)	TG	0.401348747	Down
TG (14:0/20:1/22:2)	TG	0.321364064	Down
TG (18:1/18:1/18:2)	TG	0.458430749	Down
TG (16:0/16:1/22:4)	TG	0.483735963	Down
TG (14:0/20:1/20:4)	TG	0.357699625	Down
TG (14:0/20:4/22:1)	TG	0.362040332	Down
TG (18:0/18:3/20:2)	TG	0.397535756	Down
TG (16:0/16:0/22:6)	TG	0.461920941	Down
TG (16:0/16:1/22:5)	TG	0.38943777	Down
TG (18:1/18:3/20:2)	TG	0.388550917	Down
TG (16:0/18:3/22:3)	TG	0.393336742	Down
TG (14:0/20:1/22:5)	TG	0.358915091	Down
TG (16:0/16:1/22:6)	TG	0.389759949	Down
TG (18:1/18:3/22:3)	TG	0.327154868	Down
TG (18:0/18:2/22:5)	TG	0.299294195	Down
TG (14:0/22:2/22:6)	TG	0.226285955	Down

These metabolites are enriched in some metabolic pathways including vitamin digestion and absorption, thermogenesis, lipolysis regulation in adipocytes, metabolic pathways, insulin resistance, glycerolipid metabolism, fat digestion and adsorption, and cholesterol metabolism (Figure 6B).

## 4 Discussion

The transcription of DNA into mRNA is a complex process that is essential for the genetics. A gene can produce various transcripts with varying levels of expression. Various transcripts of the ACSL1 gene in humans, pigs, mice, cattle, and other organisms have been described in various investigations. To date, five transcripts of the human ACSL1 gene, eight transcripts of the pig ACSL1 gene, six transcripts of the mouse ACSL1 gene, and four transcripts of the bovine ACSL1 gene have been published in GenBank. Two transcripts of the sheep ACSL1 gene were found by RACE and verified by Northern and Western blotting. The G–A mutation site in the cloned sequence leads to the early termination of the ACSL1 mRNA translation process. After the verification of this site on the genome, it was found that there was no SNP at this site. No SNP was found at the mutation site leading to the assumption that this base substitution was caused by RNA-editing, which is epigenetic.

Mitochondria and nuclear localization of the ACSL1 gene of sheep preadipocytes were observed through confocal laser microscopy; cytoplasmic localization was predicted as well. Various reports are also available which reveal the presence of the ACSL1 gene in the endoplasmic reticulum, nucleus, plasma membrane, mitochondria, and lipid droplets in different cell lines (Gargiulo et al., 1999; Brasaemle et al., 2004). Similarly, the ACSL1 gene was found in rat adipocytes (Sleeman et al., 1998). Furthermore, the ACSL1 gene was also found in mitochondria of PtK2 epithelial cells (Milger et al., 2006), the endoplasmic reticulum, and the cytoplasm of rat liver cells (Lewin et al., 2001). Therefore, the presence of the differentially characterized ACSL1 cannot be attributed to the specific organelle.

Since its discovery, APA has been thought to be associated with to biological functions since its discovery and may be used as a molecular marker in a variety of fields in the future. The APA phenomenon of ACSL1 has been confirmed to serve a special function in the organism. Various studies have shown that the ACSL1 gene may regulate colorectal cancer through lipid metabolism. The 3′UTR diversity of the ACSL1 gene may indicate the possibility of recurrence after treatment. Patients with genotype TT are more likely to relapse than those with genotype TC (Vargas et al., 2016). Researchers have different perspectives on the ACSL1 gene studies. Various studies believe that the ACSL1 gene can promote triglyceride synthesis (Chiu et al., 2001; Pan et al., 2010). Although some studies show that the ACSL1 gene can promote the synthesis of diglycerides and glycerophospholipids, but not the synthesis of triglycerides (Mukherjee and Yun, 2012). The present study research reveals that overexpression of ACSL1-a can boost intracellular diglyceride synthesis (Cao et al., 2020) and that the ACSL1-b gene can also promote triglyceride synthesis. The diglyceride pathway is primarily responsible for triglyceride synthesis in adipose tissue; however, there may be other interactions between the two transcripts that help to regulate triglycerides synthesis (Cao et al., 2003). In both the experiments of overexpression of ACSL1-a and knocking down of ACSL1, differential metabolites enriched in the lipolysis regulation in the adipocyte pathway were observed, which is consistent with the main function of the ACSL1 gene. Previous research has indicated that ACSL1 primarily affects the synthesis of 16–18 carbon triglycerides (Suzuki et al., 1990); however, the data show that the ACSL1 expression also influences the synthesis of 14–22 carbon triglycerides. Acylcarnitine, a byproduct of acyl-CoA oxidation, is also regulated by the ACSL1 gene. Therefore, it is believed that different transcripts of the ACSL1 gene might have different functions, but when the common region of the two transcripts knocks down the ACSL1 gene, only triglyceride synthesis is impacted, leading researchers to ignore the ACSL1 gene’s role in diglyceride synthesis. The particular reasons for these phenomena require additional investigation.

## 5 Conclusion

Based on the length differences of the sheep ACSL1 genes, two transcripts encode separate stable proteins. ACSL1-a can promote intracellular diglyceride synthesis; moreover, ACSL1-b can promote triglyceride synthesis. Lipidome sequencing revealed that when ACSL1 transcripts were knocked down, intracellular 14–22 carbon triglycerides and acylcarnitine levels were significantly reduced. Different ACSL1 transcripts perform distinct functions in sheep lipid metabolism and the combination of ACSL1-a and ACSL1-b makes the ACSL1 gene essential for triglyceride synthesis in sheep preadipocytes.

## Data Availability

The data presented in the study are deposited in the Metabolights repository, accession number MTBLS5808.
